# Body Roundness Index Trajectories and the Risk of Cancer: A Cohort Study

**DOI:** 10.1002/cam4.70447

**Published:** 2024-11-28

**Authors:** Yue Chen, Yiming Wang, Xin Zheng, Tong Liu, Chenan Liu, Shiqi Lin, Hailun Xie, Jinyu Shi, Xiaoyue Liu, Xiangming Ma, Li Deng, Shouling Wu, Hanping Shi

**Affiliations:** ^1^ Department of Gastrointestinal Surgery, Department of Clinical Nutrition, Beijing Shijitan Hospital Capital Medical University Beijing China; ^2^ Department of National Clinical Research Center for Geriatric Diseases, Xuanwu Hospital Capital Medical University Beijing China; ^3^ Key Laboratory of Cancer FSMP for State Market Regulation Beijing China; ^4^ Department of Cardiology, Kailuan General Hospital North China University of Science and Technology Tangshan China; ^5^ Department of Hepatological Surgery Kailuan General Hospital Tangshan China

**Keywords:** abdominal obesity, body roundness index, cancer, epidemiology, trajectory analysis

## Abstract

**Background:**

The body rounds index (BRI), an innovative obesity indicator integrating waist circumference (WC) and height, offers a two‐dimensional assessment of obesity. The relationship between BRI trajectories and cancer has been overlooked in previous studies. This study aims to explore the association between BRI trajectories and the incidence of cancer.

**Methods:**

This study included 42,022 participants with a median age of 48.91 years. Based on the changes in participants' BRI during the period from 2006 to 2010, three BRI trajectory patterns were identified: low‐stable, medium‐stable, and high‐stable. The primary outcome was cancer incidence and the secondary outcome was cancer‐specific deaths. The association between BRI trajectories and cancer incidence and death was explored by cox regression analysis in the total, sex‐specific and age‐specific populations, respectively. Additionally, we further investigated the relationship between BRI and site‐specific cancer incidence. Sensitivity analyses were applied to exclude interferences and ensure the stability of the results.

**Results:**

After a median follow‐up time of 11.04 years, high‐stable BRI trajectory was significantly associated with increased risk of cancer occurrence compared to low‐stable BRI trajectory. This association was more pronounced in middle‐aged men (men: HR = 1.46, 95% CI = 1.21–1.77, *p* < 0.001; age < 65: HR = 11.38, 95% CI = 1.15–1.66, *p* = 0.001). Additionally, high‐stable BRI trajectory was significantly associated with a substantial increase in the risk of site‐specific uterine cancers (HR = 4.92, 95% CI = 1.69–14.33, *p* = 0.004). Sensitivity analysis confirmed the stability of the results.

**Conclusion:**

Our study identified a significant association between a high‐stable BRI trajectory and cancer incidence, with this association being most pronounced in middle‐aged men. Moreover, the high‐stable BRI trajectory was strongly associated with uterine site‐specific cancer development. Our findings underscore the importance of implementing lifestyle modifications and monitoring BRI values and their changes to provide effective health guidance.

## 1 Introduction

The rising incidence of malignant tumors has become a pressing concern, posing a significant threat to patient survival and placing a heavy burden on healthcare systems worldwide [1, 2]. As the landscape of cancer occurrence and treatment continues to evolve, there is an urgent need for a proactive approach to risk prediction, and non‐invasive body measurements offer a promising avenue for gaining valuable insights into body composition and metabolic status [3]. Among these measures, body mass index (BMI) has garnered substantial attention as a surrogate for obesity [4]. However, mounting evidence suggests the limitations of BMI in reliably predicting cancer incidence [5, 6]. Recent studies emphasize the greater relevance of visceral fat, rather than over overall fat, in determining cancer risk [7, 8]. Therefore, it is crucial to recognize the inadequacies of BMI as a sole predictive metric in cancer risk assessment.

The body rounds index (BRI), an innovative obesity indicator integrating waist circumference (WC) and height, provides a two‐dimensional assessment of obesity. This metric adjusts for body size discrepancies by incorporating height while using WC to measure abdominal obesity. Previous research has highlighted the association between BRI and prevalent metabolic disorders like non‐alcoholic fatty liver disease and diabetes [9, 10]. Furthermore, emerging studies have suggested at a significant correlation between height and cancer incidence [11, 12]. Remarkably, the BRI amalgamates both height and abdominal obesity—two recognized risk factors for cancer. It is plausible that the potential relationship between BRI and cancer incidence may have been overlooked in prior investigations. This raises a compelling hypothesis that BRI could be associated with cancer incidence, presenting a potentially underexplored avenue for further research.

The etiology of cancer is multifaceted, often resulting from the prolonged influence of various pathogenic factors. As a result, relying solely on baseline body measurements for predicting cancer occurrence is inherently limited. Substantial changes in body size can manifest in the presence of illness, weight fluctuations, or other health conditions [13]. Compared to a single baseline measurement, using multiple measurements over time to track dynamic changes offers a more scientifically rigorous and precise approach to predicting cancer occurrence. Hence, our study's primary objective was to chart the trajectories of BRI within the population and explore the relationship between distinct BRI trajectories and cancer incidence. This approach aimed to capture the nuanced changes in BRI over time and correlate these patterns with the likelihood of cancer occurrence, fostering a comprehensive understanding of the interplay between BRI dynamics and cancer development.

## 2 Methods

### 2.1 Study Design and Populations

**FIGURE 1:**
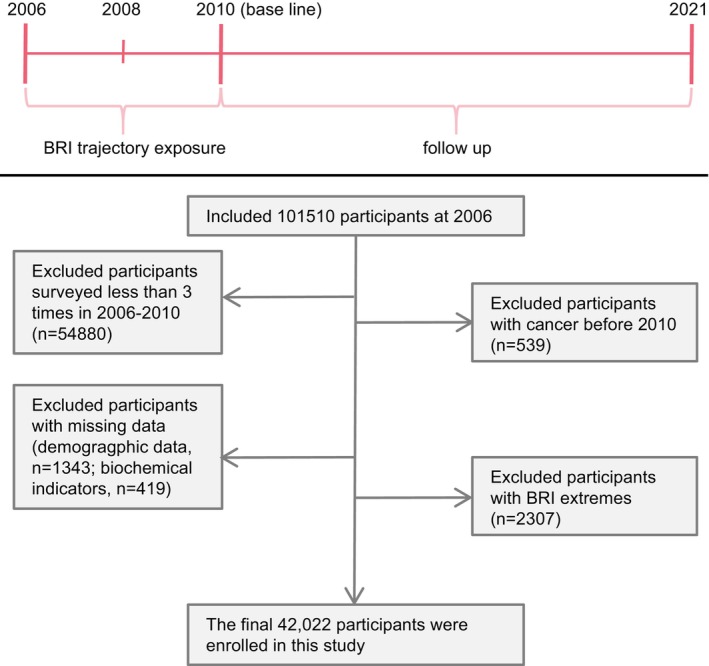
Flowchart. BRI, body roundness index.

The data used in this study were obtained from the Kailuan Cohort. The Kailuan Cohort was a prospective cohort from the Kailuan community in Tangshan, an industrial city in northern China. We invited employees (including retired employees) from the Kailuan Group to participate in 2006; 101,510 employees agreed to participate and signed a consent form. They completed questionnaires, including demographic and sociological information and lifestyle surveys. In addition, the participants underwent a blood biochemistry test in the fasting state. The life questionnaire and blood indicators were administered every 2 years. Participants who underwent fewer than two medical checkups between 2008 and 2010 or had a history of malignant neoplastic disease at baseline were excluded. The specific process of inclusion and exclusion is shown in Figure 1.

The privacy of all participants was concealed. This study adhered to the principles of the Declaration of Helsinki and was approved by the Ethics Committee of Kailuan Medical Group, Kailuan Company.

### 2.2 Variable Assessment

The height, weight, and WC of all participants were measured in the hospital by trained healthcare professionals. Participants wore light clothing and no shoes. Height was measured using a calibrated stadiometer with the participants standing straight, heels together, and eyes looking straight ahead. Weight was measured using a calibrated electronic scale. WC was measured at the midpoint between the lower margin of the last palpable rib and the top of the iliac crest at the end of a normal expiration using a non‐stretchable tape measure. On this basis, BMI was calculated by dividing weight by the square of height; and body roundness index (BRI) was calculated as follows: BRI = 364.2–365.5 × √1‐(WC/2Π)^2^/(0.5height)^2^, WC and height are both in meters [14]. Waist‐to‐weight ratio (WaWtR) was obtained by dividing WC by weight; waist‐to‐hip ratio (WaHiR) was obtained by dividing WC by hip circumference; waist‐to‐height ratio (WaHeR) was obtained by dividing WC by height; waist BMI ratio (WBR) was obtained by dividing WC by BMI.

The covariates included sex, age, education level, type of work, smoking, alcohol consumption, physical exercise, diabetes, hypertension, and dyslipidemia. This information was obtained from a 2010 questionnaire. Blood samples were collected after an overnight fast. C reactive protein (CRP) and triglyceride levels were analyzed using an autoanalyzer (Hitachi 747; Hitachi) and standardized methods.

### 2.3 Outcomes and Follow‐Up

The primary outcome of this study was cancer occurrence, and the secondary outcome was cancer‐specific deaths, which was defined as death within 5 years of cancer diagnosis. All participants were followed up from baseline (2010–2011) until cancer occurrence or death, and death information was collected from death certificates from the National Vital Statistics Service. All outcome events were counted until 2021.

### 2.4 Statistics Analysis

Continuous variables obeying normal distribution were expressed as mean ± standard deviation (SD), and a *t*‐test was applied; continuous variables not obeying normal distribution were expressed as median [interquartile range], and the rank sum test was applied. The chi‐squared test was used for categorical variables.

We used absolute values of WC as a reference to compare the predictive ability of BMI, BRI, and other relative WC metrics for cancer incidence by NRI (Net Reclassification Index); other relative WC metrics included WaWtR, WaHiR, WaHeR, and WBR. We fitted the model using the “TRAJ” program in SAS version 9.4 (SAS Institute) to group individuals with similar patterns of BRI change between 2006 and 2010. We tested models with quadratic polynomial function parameters for groups ranging from 1 to 5 and then compared the models to different functional forms with cubic, quadratic, and linear terms. The optimal number of potential trajectories was assessed using a combination of the following criteria: (1) observing improvements in the Bayesian information criterion, (2) ensuring > 5% membership in any single trajectory group, and (3) identifying visually distinct trajectories. Three groups of models were ultimately determined to be the most suitable for the BRI. In addition, the posterior predictive probabilities of the BRI for all potential categories were higher than 0.75, suggesting that our trajectory evaluations were well differentiated.

We used COX regression model to investigate the association between different BRI trajectories and cancer incidence. Where model 0 was uncorrected and model 1 adjusted for sex, age, education, type of work, smoking, alcohol consumption, physical activity, salt intake, and BMI. Model 2 was additionally corrected for CRP, triglycerides, hypertension, diabetes, and dyslipidemia based on model 1. Considering that there are differences in body size and cancer incidence by sex and age, we also stratified the analysis among different sexes, and among younger (< 65 years) and older (≧ 65 years) participants. In addition, we used the same approach to investigate the association between different BRI trajectories and cancer‐specific mortality. We used a competing risk model to compare the cumulative incidence of cancer occurrence and death among participants with different BRI trajectories.

In subgroup analyses, participants were stratified according to different lifestyles, health status, and the association between BRI trajectories and cancer incidence was studied in populations with different characteristics. Furthermore, we investigated the association between BRI trajectories and site‐specific cancers occurrence. We performed sensitivity analyses to avoid potential reverse causality after excluding individuals with outcomes in the first 2 years of follow‐up. We additionally adjusted for BRI at baseline, taking glucose‐lowering medication, taking lipid‐lowering medication, and taking antihypertensive medication, respectively, based on model 2.

All analyses used SAS (version 9.4) and R (version 4.2.3). A two sides *p*‐value < 0.05 was considered statistically significant.

## 3 Results

### 3.1 Trajectory Fitting and Population Characterization

In this study, we finally included 42,022 participants (mean age 48.91 + 11.58), including 8486 (20.19%) woman participants. The median follow‐up time was 11.04 years, and up to 2021, a total of 1875 cancer occurrences were recorded, in fact 784 cancer‐specific deaths occurred. Table S1 shows the incidence of specific cancer types.

**FIGURE 2:**
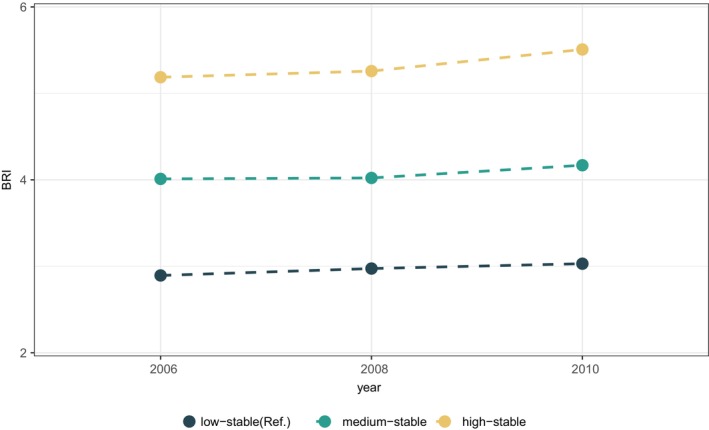
Body roundness index trajectories during 2006–2010. BRI, body roundness index.

Using absolute WC as a reference, the NRI was used to compare the predictive ability of BMI‐ and WC‐related metrics for cancer. The results showed that the BRI maximized the predictive ability of WC (NRI = 0.0577), while BMI had a lower predictive ability than WC (NRI = −0.069) (Table S2). The three best trajectories of BRI fitted to the model were low‐stable (mean BRI = 2.97, *n* = 15,752), medium‐stable (mean BRI = 4.07, *n* = 21,352), and high‐stable (mean BRI = 5.32, *n* = 4918) trajectories (Figure 2). Participants with high stable BRI trajectory tended to be older, less educated, nonsmokers, and had higher CRP and triglyceride levels (Table 1).

**TABLE 1:** Basic characteristics of participants, stratified by different body roundness index trajectories (*n* = 42,022).

	Overall	Low‐stable	Medium‐stable	High‐stable	*p*‐value
*N*	42,022	15,752	21,352	4918	
BRI (2006)	3.73 (1.03)	2.89 (0.63)	4.01 (0.76)	5.19 (0.79)	< 0.001
BRI (2008)	3.77 (0.99)	2.97 (0.57)	4.02 (0.71)	5.26 (0.78)	< 0.001
BRI (2010)	3.90 (1.06)	3.03 (0.64)	4.17 (0.74)	5.51 (0.81)	< 0.001
Mean BRI	3.80 (0.84)	2.97 (0.37)	4.07 (0.37)	5.32 (0.40)	< 0.001
Age	48.91 (11.58)	45.98 (11.53)	50.06 (11.09)	53.28 (11.56)	< 0.001
Sex, *n* (%)					
Female	8486 (20.2)	3442 (21.9)	3839 (18.0)	1205 (24.5)	< 0.001
Male	33,536 (79.8)	12,310 (78.1)	17,513 (82.0)	3713 (75.5)	
Work, *n* (%)					
Mental	2723 (6.5)	1067 (6.8)	1351 (6.3)	305 (6.2)	0.158
Physical	39,299 (79.8)	14,685 (93.2)	20,001 (93.7)	4613 (93.8)	
Education, *n* (%)					
High	8045 (19.1)	3592 (22.8)	3727 (17.5)	726 (14.8)	< 0.001
Low	33,977 (80.9)	12,160 (77.2)	17,625 (82.5)	4192 (85.2)	
Smoke, *n* (%)					
No	32,036 (76.2)	11,782 (74.8)	16,333 (76.5)	3921 (79.7)	< 0.001
Yes	9986 (23.8)	3970 (25.2)	5019 (23.5)	997 (20.3)	
Alcohol intake, *n* (%)					
No	40,088 (95.4)	15,060 (95.6)	20,331 (95.2)	4697 (95.5)	0.195
Yes	1934 (4.6)	692 (4.4)	1021 (4.8)	221 (4.5)	
Exercise, *n* (%)					
No	36,112 (85.9)	13,645 (86.6)	18,335 (85.9)	4132 (84.0)	< 0.001
Yes	5910 (914.1)	2107 (13.4)	3017 (14.1)	786 (16.0)	
Salt intake, *n* (%)					
< 6 g/d	3841 (9.1)	1574 (10.0)	1897 (8.9)	370 (7.5)	< 0.001
6–10 g/d	33,739 (80.3)	12,609 (80.0)	17,181 (80.5)	3949 (80.3)	
> 10 g/d	4442 (10.6)	1569 (10.0)	2274 (10.7)	599 (12.2)	
Comorbidities, *n* (%)					
No	33,810 (80.5)	14,058 (89.2)	16,600 (77.7)	3152 (64.1)	< 0.001
Yes	8212 (19.5)	1694 (10.8)	4752 (22.3)	1766 (35.9)	
BMI	25.15 (3.19)	22.93 (2.35)	25.90 (2.55)	29.02 (2.84)	< 0.001
WC	86.55 (8.99)	79.57 (6.50)	89.17 (6.85)	97.53 (6.72)	< 0.001
CRP	0.70 [0.27, 1.90]	0.47 [0.19, 1.23]	0.80[0.31, 2.07]	1.40[0.60, 3.90]	< 0.001
TG	1.30 [0.91, 1.99]	1.09 [0.77, 1.52]	1.42 [1.00, 2.17]	1.68 [1.17, 2.49]	< 0.001

*Note:* Continuous variables were presented as mean ± standard deviation (SD). Continuous variables that were not normally distributed were expressed as the median (interquartile range). Categorical variables were presented as numbers and percentages. Differences in normally and non‐normally distributed baseline characteristics were compared using the chi‐square test or *t*‐test and Wilcoxon rank sum test, respectively. Participants were considered to have co‐morbidities as long as they had hypertension, diabetes, or dyslipidemia.

Abbreviations: BMI, body mass index; BRI, body roundness index; CRP, C reactive protein; TG, triglyceride; WC, waist circumference.

### 3.2 Association of BRI Trajectories With Cancer Risk

We recorded 1875 cancer occurrences during the follow‐up period, with the highest cancer incidence of 5.67% in the high‐stable BRI trajectory group. The hazard ratio (HR) for cancer occurrence was 1.13 (95% CI: 1.01–1.27) in the medium‐stable group and 1.35 (95% CI: 1.14–1.60) in the high‐stable group compared to the low‐stable group of the BRI trajectory. A significant association between high‐stable BRI trajectory and increased cancer risk remained in men, but no significant association was observed in women after correction for confounding factors (men: adjusted HR = 1.46, 95% CI: 1.21–1.77, *p* < 0.001; women: adjusted HR = 1.04, 95% CI: 0.72–1.50, *p* = 0.822). In addition, when we used 65 years as a cutoff to distinguish between younger and older adults, we found that the association between high‐stable BRI trajectory and cancer risk was more pronounced in those younger than 65 years of age (age < 65 years: adjusted HR = 1.38, 95% CI: 1.15–1.66, *p* = 0.001; age ≧ 65 years: adjusted HR = 1.24, 95% CI: 0.85–1.81, *p* = 0.001) (Table 2).

**TABLE 2:** Association between body roundness index trajectories and risk of cancer incidence, stratified by sex and age.

BRI trajectories (all)	Low‐stable	Moderate‐stable		High‐stable	
*Overall*					
Participants subjects, *n*	15,752	21,352		4918	
Cancer cases, *n* (%)	625 (3.97%)	971 (4.55%)		279 (5.67%)	
Mean BRI	2.967424	4.067487		5.317628	
		HR (95% CI)	*p*‐value	HR (95% CI)	*p*‐value
Model 0	Reference	1.17 (1.05, 1.29)	0.003	1.50 (1.31, 1.72)	0.001
Model 1	Reference	1.14 (1.02, 1.28)	0.022	1.39 (1.18, 1.64)	< 0.001
Model 2	Reference	1.13 (1.01, 1.27)	0.035	1.35 (1.14, 1.60)	0.001

BRI trajectories (stratified by sex)	Low‐stable	Moderate‐stable		High‐stable	
*Man*					
Participants subjects, *n*	12,310	17,513		3713	
Cancer cases, *n*	502	791		210	
Mean BRI	2.985549	4.062914		5.299903	
		HR (95% CI)	*p*‐value	HR (95% CI)	*p*‐value
Model 0	Reference	1.13 (1.01, 1.26)	0.037	1.46 (1.24, 1.71)	< 0.001
Model 1	Reference	1.18 (1.04, 1.34)	0.009	1.51 (1.25, 1.82)	0.001
Model 2	Reference	1.17 (1.03, 1.33)	0.015	1.46 (1.21, 1.77)	< 0.001
*Woman*					
Participants subjects, *n*	3442	3839		1205	
Cancer cases, *n*	123	180		69	
Mean BRI	2.902604	4.088346		5.372244	
		HR (95% CI)	*p*‐value	HR (95% CI)	*p*‐value
Model 0	Reference	1.33 (1.06, 1.67)	0.015	1.66 (1.23, 2.22)	0.001
Model 1	Reference	1.05 (0.81, 1.36)	0.731	1.11 (0.78, 1.58)	0.555
Model 2	Reference	1.03 (0.79, 1.34)	0.836	1.04 (0.72, 1.50)	0.822

BRI trajectories (stratified by age)	Low‐stable	Moderate‐stable		High‐stable	
*Age < 65*					
Participants subjects, *n*	14,758	19,204		4103	
Cancer cases, *n*	560	808		225	
Mean BRI	2.96129	4.058859		5.309105	
		HR (95% CI)	*p*‐value	HR (95% CI)	*p*‐value
Model 0	Reference	1.12 (1.01, 1.25)	0.039	1.48 (1.27, 1.73)	< 0.001
Model 1	Reference	1.12 (0.99, 1.26)	0.077	1.45 (1.21, 1.74)	< 0.001
Model 2	Reference	1.09 (0.97, 1.24)	0.153	1.38 (1.15, 1.66)	0.001
*Age ≧ 65*					
Participants subjects, *n*	994	2148		815	
Cancer cases, *n*	65	163		54	
Mean BRI	3.058497	4.14462		5.360535	
		HR (95% CI)	*p*‐value	HR (95% CI)	*p*‐value
Model 0	Reference	1.18 (0.89, 1.57)	0.258	1.04 (0.73, 1.49)	0.828
Model 1	Reference	1.26 (0.92, 1.71)	0.144	1.18 (0.77, 1.80)	0.443
Model 2	Reference	1.30 (0.97, 1.75)	0.077	1.24 (0.85, 1.81)	0.251

*Note:* Data are presented as hazard ratios (95% confidence intervals). Model 0: unadjusted; Model 1: adjusted for sex, age, education, work, smoke, alcohol consumption, physical exercise, BMI (In gender‐stratified analysis, sex was not adjusted, and in age‐stratified analysis, age was not adjusted); Model 2: adjusted for CRP, TG, diabetes, hypertension, dyslipidemia base on model 1.

Abbreviations: 95% CI, 95% confidence intervals; BRI, body roundness index; HR, hazard ratio.

**FIGURE 3:**
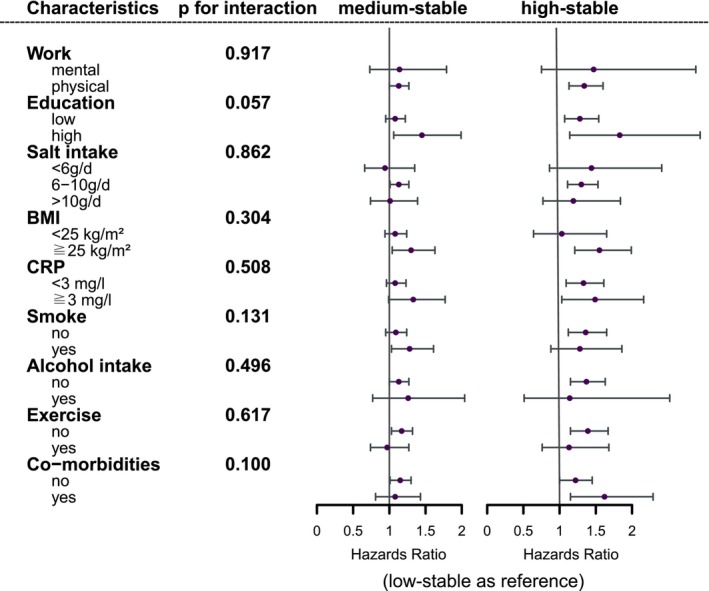
Association of body mass index trajectories and cancer occurrence in participants with different characteristics. BMI, body mass index; CRP, C reactive protein.

In addition, we conducted subgroup analyses among participants with different characteristics. The results showed that high stable BRI trajectory was positively associated with an increased risk of cancer in populations with almost all characteristics. However, this association was not significant in participants who were engaged in mental work, had a low BMI, smoked, and drank alcohol (Figure 3).

Furthermore, we investigated the association between medium‐ and high‐stable BRI trajectories and site‐specific cancer incidence, using the low‐stable BRI trajectory group as a reference. The results found that both medium‐ and high‐stable BRI trajectories were significantly associated with an increased risk of hepatobiliary cancers (medium‐stable: HR = 1.67, 95% CI: 1.22–2.49, *p* = 0.013; high‐stable: HR = 1.86, 95% CI: 1.03–3.36, *p* = 0.040). There were significant associations between high‐stable BRI trajectory and the incidence of uterine, cervical, and prostate cancers (uterine and cervical: HR = 4.92, 95% CI: 1.69–14.33, *p* = 0.004; prostate: HR = 2.50, 95% CI: 1.04–5.98, *p* = 0.040). In addition, the results revealed a significant association between medium‐stable BRI trajectory and the incidence of hematologic neoplasms (HR = 1.74, 95% CI: 1.16–6.74, *p* = 0.028) (Table 3).

**TABLE 3:** Association between BRI trajectories and site‐specific cancers incidence.

	Low‐stable	Medium‐stable		High‐stable	
		HR (95% CI)	*p*‐value	HR (95% CI)	*p*‐value
Head and neck	Reference	0.72 (0.48,1.08)	0.111	0.96 (0.51,1.81)	0.900
Esophagus	Reference	1.43 (0.79,2.59)	0.238	1.35 (0.53,3.65)	0.553
Gastrointestinal	Reference	1.13 (0.76,1.68)	0.553	1.65 (0.92,2.96)	0.090
Colorectal	Reference	1.02 (0.75,1.39)	0.884	1.16 (0.72,1.84)	0.544
Liver and gallbladder	Reference	1.67 (1.22,2.49)	0.013	1.86 (1.03,3.36)	0.040
Pancreas	Reference	1.23 (0.62,2.53)	0.573	0.56 (0.16,1.98)	0.371
Lung	Reference	1.13 (0.91,1.40)	0.259	1.15 (0.82,1.62)	0.411
Breast	Reference	1.01 (0.62,1.64)	0.966	1.60 (0.82,3.09)	0.167
Uterus and cervix	Reference	1.48 (0.65,3.37)	0.356	4.92 (1.69,14.33)	0.004
Ovary	Reference	0.81 (0.16,4.16)	0.800	1.65 (0.23,11.87)	0.621
Prostate	Reference	0.96 (0.48,1.90)	0.898	2.50 (1.04,5.98)	0.040
Kidney	Reference	1.59 (0.87,2.91)	0.135	2.15 (0.94,4.94)	0.071
Urinary bladder	Reference	1.08 (0.58,2.01)	0.813	0.91 (0.35,2.38)	0.841
Hematology^a^	Reference	2.74 (1.16,6.74)	0.028	2.69 (0.81,8.96)	0.108

^a^ Tumors of the hematology include lymphoma and leukemia.

### 3.3 Association of BRI Trajectories With Cancer‐Specific Mortality

The results of the COX regression analysis showed a significant association between BRI trajectories and cancer‐specific mortality. Compared to the low‐stable BRI trajectory group, the medium‐ and high‐stable groups had a 20% and 31% increased cancer risk, respectively (medium‐stable: HR = 1.20, 95% CI: 1.01–1.43, *p* = 0.041; high‐stable: HR = 1.31, 95% CI: 1.00–1.71, *p* = 0.046). Similarly, such association remained significant among men and participants younger than 65 years of age and was not significant among women and participants older than 65 years of age (men: HR = 1.37, 95%CI: 1.03–1.81, *p* = 0.029; age < 65 years: HR = 1.38, 95% CI: 1.15–1.66, *p* = 0.001) (Table S3). Competing risk plot showed that the risk of death was higher than cancer occurrence in the medium‐stable and high‐stable groups of BRI trajectories and that the risk of death was lowest in the low‐stable group (Figure S1).

### 3.4 Sensitivity Analyses

Sensitivity analysis performed by excluding the cases during the first 2 years showed a positive and robust association between BRI trajectory and cancer risk. Further, we additionally adjusted for BRI at baseline, taking antihypertensive, hypoglycemic, and lipid‐lowering medications, respectively, on top of model 2. The results suggested that these factors did not affect the relationship between BRI trajectory and cancer risk (Table 4).

**TABLE 4:** Sensitivity analysis.

	HR (95% CI)	*p*‐value
*Exclusion of participants with outcomes in the first 2 years of follow‐up*		
Low‐stable	Reference	
Medium‐stable	1.17 (1.03, 1.32)	0.014
High‐stable	1.40 (1.17, 1.69)	< 0.001
*Additional adjust for base line BRI*		
Low‐stable	Reference	
Medium‐stable	1.13 (0.98, 1.30)	0.081
High‐stable	1.31 (1.03, 1.66)	0.026
*Additional adjust for antihyperlipidemic drug*		
Low‐stable	Reference	
Medium‐stable	1.16 (1.03, 1.32)	0.016
High‐stable	1.39 (1.16, 1.67)	< 0.001
*Additional adjust for antihypertensive drug*		
Low‐stable	Reference	
Medium‐stable	1.17 (1.03, 1.32)	0.015
High‐stable	1.40 (1.16, 1.68)	< 0.001
*Additional adjust for antihyperglycemic drug*		
Low‐stable	Reference	
Medium‐stable	1.17 (1.03, 1.32)	0.014
High‐stable	1.40 (1.17, 1.69)	< 0.001

Abbreviation: BRI, body roundness index.

## 4 Discussion

In this study, from 2006 to 2010, we identified three distinct BRI trajectories among 42,022 participants, categorized as low‐stable, medium‐stable, and high‐stable types. We observed a significant association between three BRI trajectories and the risk of cancer development. Compared to the low‐stable group, both the moderate‐stable and high‐stable BRI trajectories were significantly associated with an increased risk of cancer incidence. This association was particularly pronounced in men and in young and middle‐aged adults below 65 years of age.

There is growing evidence indicating a close association between obesity and the occurrence and progression of various cancers [15]. When the body becomes obese, hormones secreted by adipose tissue change, especially estrogen, insulin, and growth hormone [16, 17, 18]. These hormones stimulate cell growth and proliferation, increasing the risk of cancer [19, 20]. Additionally, in the state of obesity, adipose tissue releases various cytokines, such as tumor necrosis factor‐α (TNF‐α) and interleukin 6 (IL‐6), which play a role in chronic inflammation [21]. Long‐term chronic inflammation may lead to cellular damage and DNA mutations that increase cancer risk [22]. Currently, most assessments of obesity are based on BMI, but BMI lacks the ability to assess fat distribution [23]. A multinational cohort study found that high BMI with low abdominal circumference was not associated with cancer risk [3]. Therefore, it is essential to take WC into consideration when studying obesity and cancer. Previous studies have found that abdominal obesity is a more important causative risk factor for cancer than obesity [24]. Abdominal fat, especially visceral fat, is more active than subcutaneous fat in secreting hormones and bioactive substances. These molecules may affect lipid metabolism, inflammatory responses, and cellular signaling pathways, thereby increasing the risk of developing diseases [25].

As a novel obesity indicator, there has been limited research on the association between BRI and cancer. Gao et al. examined BRI trajectories and colorectal cancer risk, but observed an association only in a cross‐sectional setting [26]. Long‐term changes in body size may have different effects on cancer development. In our prospective cohort study of adults (2006–2010, with biennial follow‐up), we used BRI trajectories as a study variable. A higher BRI implies a body shape closer to round, indicating a larger WC and shorter height. Previous studies have shown significant associations between different BRI trajectories and increased risks of all‐cause mortality and cardiovascular mortality [27, 28]. Our findings suggest that participants with high and stable BRI trajectories are at greater risk of developing cancer. Higher BRI has long been recognized as a metabolic risk factor associated with metabolic syndrome [29]. This study, for the first time in a large prospective cohort, reveals a significant association between different BRI trajectories and cancer incidence. The association between BRI and cancer incidence is multifaceted. On one hand, this index reflects the accumulation of abdominal fat. The relationship between abdominal fat and cancer has been widely studied, with specific biological mechanisms suggesting that visceral fat is more metabolically active than subcutaneous fat, secreting more pro‐inflammatory factors and hormones, thereby increasing cancer risk [30]. Furthermore, BRI reflects the sphericity of the body. Unlike WC, which measures a single dimension, BRI takes into account the overall shape of the body. Our results indicate that BRI outperforms WC in predicting cancer incidence (NRI = 0.0577). Previous study has shown that different body shapes are associated with varying cancer risks [3]. The specific mechanisms linking body shape and cancer incidence warrant further investigation in future research.

Notably, there were sex differences in the association between BRI trajectories and cancer. We found that the association between BRI trajectory and risk of cancer incidence or death was more significant in men. However, in women, the association lost significance after adjusting for confounding factors. Despite this, we observed an interesting phenomenon in which the BRI trajectories were significantly associated with a substantial increase in the risk of uterine cancers in women. Previous research indicates that obesity is associated with an increase in circulating estradiol levels, and adipose tissue can significantly contribute to the estrogen circulating pool [31]. Additionally, the liver is the primary organ for estrogen metabolism, and obesity may disrupt the normal metabolism of estrogen in the liver, leading to a further increase in serum estrogen levels [32, 33]. In addition, estradiol levels are higher in adipose tissue of obese women compared to men [34]. These evidences suggest a hyperestrogenic state in obese women. Overexposure to estrogen increases the risk of endometrial cancer [35]. Padilla‐Banks et al. demonstrated that widespread activation of the Wnt/β‐catenin signaling pathway in estrogen‐exposed uterine epithelial cells, which, in turn, significantly increased the inflammatory signaling pathway and cellular oxidative stress, contributing to the development of cancer [36]. These evidences may account for the association of high stable BRI trajectories with a high risk of uterine site‐specific cancers.

In addition, we found that the association between BRI trajectories and the risk of cancer is more significant in middle‐aged and young adults compared to individuals aged over 65. In young and middle‐aged populations, weight loss (especially abdominal obesity) is beneficial to health. However, weight loss may not improve the survival status of older individuals, particularly in the presence of comorbidities [37]. Nevertheless, caution should still be exercised regarding visceral obesity. With advancing age, the redistribution of fat, the formation of new visceral fat, and the resulting metabolic disturbances, such as inflammation, insulin resistance, and sarcopenic obesity, may contribute to adverse outcomes [38].

In our study, conducted in a community‐based population, we observed a significant association between BRI trajectory and the risk of cancer and mortality, especially in young and middle‐aged men. BRI trajectory has a positive impact on the prevention of uterine site cancers in women. One strength of this study is its large‐scale prospective cohort design, involving multiple physical examinations and follow‐ups for participants, closely tracking changes in their body shape parameters. Additionally, the broad age range of participants allows for the observation of body shape variations across different age groups. Our study also has some limitations. First, the Kailuan cohort included participants from the Kailuan Coal Mining Group, and there were more men than women in the cohort. This makes it a limitation to study some woman‐specific diseases. Secondly, in our study, we did not observe subgroups with drastic changes in BRI trajectories, which might be attributed to the relatively short exposure period for observing BRI changes. Lastly, the majority of participants in the cohort are from the northern regions of China, and the study does not provide evidence regarding whether the association between BRI trajectories and cancer holds consistently across different ethnic populations. In future studies, BRI trajectories can be used in conjunction with other patient health information to develop personalized risk assessment tools that can help physicians make medical decisions and improve patient outcomes.

## 5 Conclusion

Our study found that high stable BRI trajectories were significantly associated with an increased risk of cancer development and mortality, especially in young and middle‐aged men. Furthermore, the high‐stable BRI trajectory showed a significant relationship with a higher incidence of uterine cancer. This study offers critical recommendations for clinicians and patients on body shape management. For public health professionals, our findings underscore the importance of implementing lifestyle modifications and monitoring BRI values and their changes to provide effective health guidance.

## Author Contributions

**Yue Chen:** conceptualization (equal), formal analysis (equal), software (equal), writing – original draft (equal). **Yiming Wang:** data curation (equal), formal analysis (equal), methodology (equal), writing – review and editing (equal). **Xin Zheng:** data curation (equal), investigation (equal), methodology (equal), writing – review and editing (equal). **Tong Liu:** data curation (equal), methodology (equal), software (equal). **Chenan Liu:** investigation (equal), methodology (equal). **Shiqi Lin:** methodology (equal), software (equal). **Hailun Xie:** resources (equal), software (equal). **Jinyu Shi:** resources (equal), software (equal). **Xiaoyue Liu:** data curation (equal), investigation (equal). **Xiangming Ma:** data curation (equal), investigation (equal), resources (equal). **Li Deng:** methodology (equal), supervision (equal). **Shouling Wu:** conceptualization (equal), data curation (equal), investigation (equal), project administration (equal). **Hanping Shi:** funding acquisition (equal), supervision (equal), validation (equal), writing – review and editing (equal).

## Ethics Statement

This study followed the Helsinki declaration. All participants signed an informed consent form. Trial registration: Kailuan study, ChiCTR‐TNRC‐11001489. Registered August 24, 2011‐Retrospectively registered, http://www.chictr.org.cn/showprojen.aspx?proj=8050.

## Consent

Informed consent was received from all participants.

## Conflicts of Interest

The authors declare no conflicts of interest.

## Declaration of Generative AI in Scientific Writing

In the analysis and writing of this study, AI was used only for language polishing.

## Supporting information


Data S1:

## Data Availability

The datasets generated during and/or analyzed during the current study are available from the corresponding author on reasonable request.
