# Supplementary material for: Body Roundness Index Trajectories and the Risk of Cancer: A Cohort Study
Source: Cancer Med. 2024 Nov 28;13(23):e70447. doi: 10.1002/cam4.70447 (PMC11602755; doi:10.1002/cam4.70447)
Supplement: Supplementary file 1 — Data S1: [file CAM4-13-e70447-s001.docx]

**Body Roundness Index Trajectories and the Risk of Cancer: A Cohort Study**

**Table. S1** Cancer incidence among participants during 2010-2021, stratified by body roundness trajectories

|  | **Overall** | **Low-stable** | **Medium-stable** | **High-stable** |
| --- | --- | --- | --- | --- |
| **Site-specific cancer** | **Case, n** | **Case, n** | **Case, n** | **Case, n** |
| head and neck | 140 | 63 | 59 | 18 |
| esophagus | 67 | 22 | 38 | 7 |
| gastrointestine | 154 | 51 | 78 | 25 |
| colorectum | 255 | 90 | 131 | 34 |
| liver and gallbladder | 159 | 43 | 93 | 23 |
| pancreas | 47 | 14 | 29 | 4 |
| lung | 513 | 179 | 271 | 63 |
| breast | 110 | 37 | 53 | 20 |
| uterus and cervix | 39 | 12 | 17 | 10 |
| ovray | 11 | 3 | 5 | 3 |
| prostate | 58 | 18 | 24 | 16 |
| kidney | 76 | 19 | 43 | 14 |
| uninary bladder | 62 | 20 | 34 | 8 |
| hematology* | 42 | 7 | 28 | 7 |

**Notes：***Tumors of the hematology include lymphoma and leukemia.

**Table. S2** Prediction of cancer occurrence by baseline anthropometric measure

| **Compared with WC** | | |
| --- | --- | --- |
|  | **NRI** | **95%CI** |
| **BRI** | 0.0577 | 0.0025, 0.0778 |
| **BMI** | -0.069 | -0.095, -0.011 |
| WaWtR | 0.0032 | -0.0261, 0.0308 |
| WaHiR | -0.0272 | -0.0685, -0.0016 |
| WaHeR | 0.009 | -0.029, 0.017 |
| **WBR** | -0.0097 | -0.0225, 0.0213 |

**Notes:** WC, waist circumference; BRI, body round index; BMI, body mass index; WaWtR, waist-weight ratio; WaHiR, waist-hipline ratio; WaHeR, waist-height ratio; WBR, waist-BMI ratio; NRI, net reclassification index; CI, confidence interval.

**Table. S3** Association between body roundness index trajectories and risk of cancer death, stratified by sex and age

| **BRI trajectories (all)** | | | | | |
| --- | --- | --- | --- | --- | --- |
| **overall** | **low-stable** | **moderate-stable** | | **high-stable** | |
| Participants subjects, *n* | 15752 | 21352 |  | 4918 |  |
| Death cases,*n* | 250 | 423 |  | 111 |  |
| Mean BRI | 2.967424 | 4.067487 |  | 5.317628 |  |
| HR (95%CI) |  |  |  |  |  |
| model 0 | Reference | 1.26 (1.08,1.48) | 0.003 | 1.47 (1.18,1.84) | 0.001 |
| model 1 | Reference | 1.21 (1.02,1.44) | 0.03 | 1.37 (1.06,1.78) | 0.018 |
| model 2 | Reference | 1.20 (1.01,1.43) | 0.041 | 1.31 (1.00,1.71) | 0.046 |
| **BRI trajectories (stratified by sex)** | | | | | |
| **man** | **low-stable** | **moderate-stable** | | **high-stable** | |
| Participants subjects, *n* | 12310 | 17513 |  | 3713 |  |
| Death cases,*n* | 236 | 385 |  | 98 |  |
| Mean BRI | 2.985549 | 4.062914 |  | 5.299903 |  |
| HR (95%CI) |  |  |  |  |  |
| model 0 | Reference | 1.16 (0.99,1.36) | 0.074 | 1.43 (1.13,1.81) | 0.003 |
| model 1 | Reference | 1.20 (1.01,1.44) | 0.042 | 1.41 (1.07,1.86) | 0.014 |
| model 2 | Reference | 1.20 (1.00,1.44) | 0.051 | 1.37 (1.03,1.81) | 0.029 |
| **woman** | **low-stable** | **moderate-stable** | | **high-stable** | |
| Participants subjects, *n* | 3442 | 3839 |  | 1205 |  |
| Death cases,*n* | 14 | 38 |  | 13 |  |
| Mean BRI | 2.902604 | 4.088346 |  | 5.372244 |  |
| HR (95%CI) |  |  |  |  |  |
| model 0 | Reference | 2.47 (1.34,4.55) | 0.004 | 2.73 (1.28,5.80) | 0.009 |
| model 1 | Reference | 1.40 (0.71,2.75) | 0.333 | 1.06 (0.44,2.53) | 0.905 |
| model 2 | Reference | 1.30 (0.66,2.59) | 0.451 | 0.88 (0.35,2.19) | 0.782 |
| **BRI trajectories (stratified by age)** | | | | | |
| age<65 | **low-stable** | **moderate-stable** | | **high-stable** | |
| Participants subjects, *n* | 14758 | 19204 |  | 4103 |  |
| Death cases,*n* | 206 | 317 |  | 72 |  |
| Mean BRI | 2.96129 | 4.058859 |  | 5.309105 |  |
| HR (95%CI) |  |  |  |  |  |
| model 0 | Reference | 1.19 (1.00,1.42) | 0.052 | 1.28 (0.98,1.67) | 0.071 |
| model 1 | Reference | 1.12 (0.99,1.26) | 0.077 | 1.45 (1.21,1.74) | <0.001 |
| model 2 | Reference | 1.09 (0.97,1.24) | 0.153 | 1.38 (1.15,1.66) | 0.001 |
| age≧65 | **low-stable** | **moderate-stable** | | **high-stable** | |
| Participants subjects, *n* | 994 | 2148 |  | 815 |  |
| Death cases,*n* | 44 | 106 |  | 39 |  |
| Mean BRI | 3.058497 | 4.14462 |  | 5.360535 |  |
| HR (95%CI) |  |  |  |  |  |
| model 0 | Reference | 1.13 (0.80,1.61) | 0.492 | 1.11 (0.72,1.71) | 0.639 |
| model 1 | Reference | 1.26 (0.92,1.71) | 0.144 | 1.18 (0.77,1.8) | 0.443 |
| model 2 | Reference | 1.32 (0.97,1.81) | 0.077 | 1.29 (0.84,1.98) | 0.251 |

**Notes:** Data are presented as hazard ratios (95% confidence intervals).

Model 0: unadjusted;

Model 1: adjusted for sex, age, education, work, smoke, alcohol consumption, physical exercise, BMI (In gender-stratified analysis, sex was not adjusted, and in age-stratified analysis, age was not adjusted);

Model 2: adjusted for CRP, TG, diabetes, hypertension, dyslipidemia base on model 1.

BRI, body roundness index; HR, hazard ratio; 95% CI, 95% confidence intervals.

**Figure. S1** Competing risk relationships between BRI trajectories and cancer occurrence and death


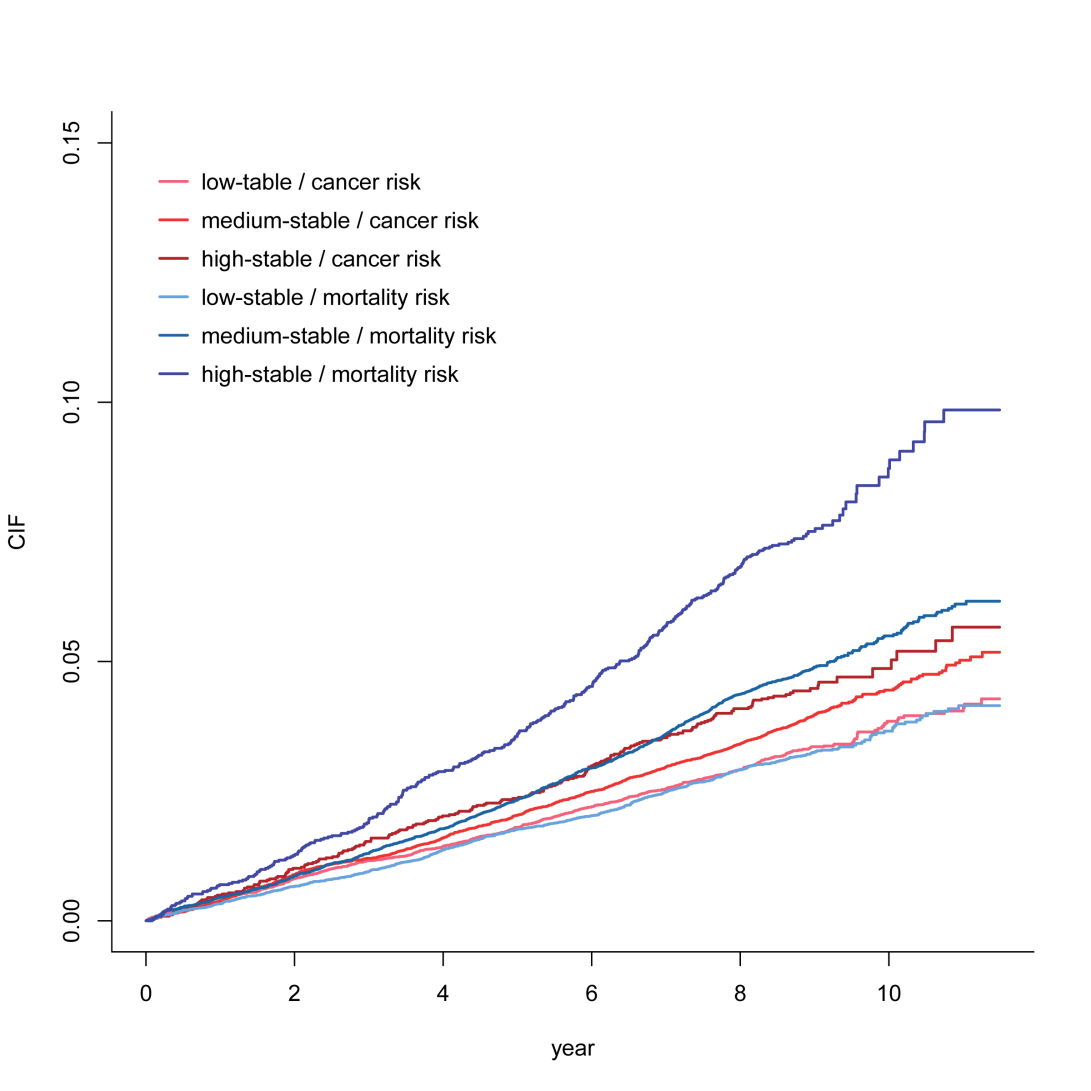


**Note:** CIF, cumulative incidence function
